# Economic evaluations of human milk for very preterm infants: a systematic review

**DOI:** 10.3389/fped.2025.1534773

**Published:** 2025-03-20

**Authors:** Anfeng Lu, Peilu Huang, Xin Guo, Li Zhu, Lei Bi, Ruirui Xing, Zhangbin Yu, Hong Tang, Guosheng Huang

**Affiliations:** ^1^ Department of Neonatology, Qinzhou Maternity and Child Health Care Hospital, Qinzhou, Guangxi, China; ^2^ Department of Neonatology, Longgang District Maternity & Child Healthcare Hospital of Shenzhen City (Longgang Maternity and Child Institute of Shantou University Medical College), Shenzhen, Guangdong, China; ^3^ Department of Neonatology, Shenzhen People’s Hospital (The Second Clinical Medical College, Jinan University, The First Affiliated Hospital, Southern University of Science and Technology), Shenzhen, Guangdong, China; ^4^ Department of Neonatology, Shenzhen Yantian District People’s Hospital, Shenzhen, Guangdong, China

**Keywords:** human milk, preterm infants, cost-effectiveness, economic evaluation, health outcomes

## Abstract

**Background:**

Very preterm infants are highly vulnerable to complications, imposing a significant economic burden on healthcare systems. Human milk has protective effects on these infants, but there is no systematic review on its economic impact.

**Objective:**

We conducted a comprehensive review of studies assessing the economic evaluations of human milk for very preterm infants.

**Methods:**

Our literature search covered PubMed, Embase, the Cochrane Library, and Web of Science. Two reviewers independently extracted data on economic evaluations and assessed study quality using the Pediatric Quality Appraisal Questionnaire (PQAQ).

**Results:**

Fourteen studies of moderate quality, conducted in the United States, Germany, and Canada, met the inclusion criteria. However, the studies analyzed had notable variations and shortcomings. The majority of these studies (*n* = 11) performed economic evaluations from a healthcare system perspective, utilizing cost-consequence analysis (*n* = 6) up to the point of neonatal discharge (*n* = 11). All human milk interventions indicated cost-effective or cost saving results; only a minority included discounting (*n* = 2).

**Conclusion:**

This systematic review suggests that economic evaluation of human milk for very preterm infants is an expanding area of research. Human milk for very preterm infants offers substantial economic advantages during neonatal intensive care unit hospitalization. Standardized and high-quality studies are needed to determine the cost-effectiveness of human milk for very preterm infants in the future.

**Systematic Review Registration:**

https://www.crd.york.ac.uk/PROSPERO, identifier (CRD42024539574).

## 1 Introduction

Worldwide from 2010 to 2020, approximately 15% of all preterm births occurred at less 32 weeks of gestation (1). They have a substantial economic impact on healthcare systems, largely due to the cost of initial hospitalization (2). There is a negative correlation between gestational age and the median cost of neonatal intensive care unit (NICU) hospitalization (3). Though very preterm infants (VPIs, gestational age < 32 weeks) make up only 1.4% of total births, they account for 36.5% of newborn care costs, making them among the most expensive hospitalized patients (4). VPIs also are particularly susceptible to developing serious morbidities associated with prematurity, such as bronchopulmonary dysplasia (BPD), necrotizing enterocolitis (NEC), late-onset sepsis (LOS), retinopathy of prematurity (ROP), intraventricular hemorrhage (IVH), and periventricular leukomalacia (PVL) (5, 6), and having substantially higher risks of adverse outcomes. Meanwhile, these morbidities significantly increase the risk of mortality in those infants, imposing a significant burden on families, healthcare programs, and educational systems throughout childhood (7). Therefore, strategies aimed at reducing the incidence, severity, and risk of these preventable morbidities during initial NICU hospitalization are crucial from both clinical and economic perspectives.

Human milk (HM) is abundant in nutrients and protective immunomodulatory components (e.g., bioactive enzymes and immune cells) and is the “gold standard” for preterm infant nutrition. It adapts dynamically to preterm infant needs to fully support healthy infant development for the first six months of life, and has the dynamic ability to be optimally matched to the evolving stages of preterm infants' immune system development (8). Multiple studies have demonstrated the effectiveness of HM in reducing the incidence, severity, and/or risk of prematurity-related morbidities, including NEC (9, 10), LOS (9, 11), BPD (12, 13) and ROP (14) during the NICU hospitalization at critical post-birth exposure periods, and the impact on these neonatal complications in a dose-dependent manner (15, 16). Accordingly, the American Academy of Pediatrics has released a statement on breastfeeding and has recommended all preterm infants should receive HM (17). However, current breastfeeding rates among VPIs for exclusive mother's own milk (MOM) at discharge is still far from satisfactory, ranging from 41.18% to 52% (1, 18). It is *a priori*ty for global health policy to increase very preterm infants' breastfeeding rates in NICU.

However, HM feeding in the NICU incurs costs because it requires an infrastructure within each NICU focused on acquiring and feeding HM. Meanwhile, the healthcare system has limited resources and must allocate them cost-effectively. Economic evaluations compare the costs and benefits of different feeding strategies to estimate which is more likely to be cost-effective (i.e., the lowest cost per unit of benefit) in the NICU. This can provide evidence for policymakers to allocate these limited resources and inform their decisions. By identifying preventable morbidities that are costly and have long-term health consequences for VPIs, society can prioritize interventions based on their impact on reducing the incidence and severity of these morbidities, giving the highest priority to those with the greatest benefit relative to cost.

Prior research has estimated the cost savings associated with feeding HM to VPIs by reducing the incidence of prematurity-related complications (19, 20). Johnson et al. (19–21) and Patel et al. (15, 16) have conducted extensive research on the cost-effectiveness of HM for VPIs. Although there is increasing research exploring the economic analysis of HM for VPIs, there is no systematic review. The aim of this systematic review is to identify, consolidate, and critically appraise published evidence on the economic evaluations of HM for VPIs, to enhance breastfeeding rates among VPIs in the NICU and improve health outcomes for this population.

## 2 Methods

### 2.1 Registration

This systematic review was reported according to the Preferred Reporting Items for Systematic reviews and Meta-Analyses (PRISMA) 2020 checklist PRISMA guidelines (22). A comprehensive review protocol, which includes objectives, eligibility criteria, information sources, and search strategies, has been registered with the International Prospective Register of Systematic Reviews (registration number: CRD42024539574). As a systematic review of published studies, ethical approval was not required nor sought.

### 2.2 Inclusion and exclusion criteria

We included cohort, randomized controlled trials, and case-control studies that examined economic evaluations of HM for VPIs. We excluded reviews, case reports, protocols, comments, case series, expert opinion and editorials. We reviewed the reference lists of the included studies to identify additional studies. The participants, interventions, comparison, outcomes and study design (PICOS) of our studies are listed below.

Participants (P): We included preterm infants born with a gestational age of less than 32 weeks and/or birth weight less than 1,500 g who were admitted to the neonatal ward. We excluded all infants known to have congenital malformations or chromosomal disorders.

Intervention (I): VPIs were fed with HM (mother's own milk, donor milk).

Comparator (s)/control (C): no restrictions on comparator (s). All alternative infant feeding options.

Outcomes (O): Our study examined clinical outcomes related to preterm birth, including NEC, BPD, LOS, ROP, and length of hospital stay. Additionally, we investigated health economic outcomes, including the initial hospitalization cost and the additional cost associated with prematurity-related complications like NEC, LOS, BPD, and ROP. This analysis included cost savings, incremental cost-effectiveness ratio (ICER), Life-Year-Gained, and the additional benefits and costs of interventions aimed at reducing the occurrence and severity of these complications.

Study design (S): Studies have reported various forms of economic evaluation concerning the use of HM to reduce complications in VPIs, including cost analyses, cost-effectiveness analyses (CEA), cost-utility analyses (CUA), cost-benefit analyses (CBA), cost-consequence analysis (CCA), and decision analytic models. CEA compares the costs and outcomes of two or more interventions, where outcomes are measured in natural units (e.g., life-years gained, cases prevented). Its results are often expressed as an ICER, which quantifies the additional cost per additional unit of effect. CUA is a specialized form of CEA that incorporates both the quantity and quality of life into its outcome measurement. Outcomes are typically expressed in quality-adjusted life years (QALYs) or disability-adjusted life years (DALYs), allowing for the comparison of interventions across different health conditions. CBA evaluates interventions by converting both their costs and benefits into monetary terms. This method facilitates a direct comparison of costs and benefits, enabling the calculation of net benefits or benefit-cost ratios to determine whether an intervention's benefits outweigh its costs. CCA presents costs and a variety of outcomes (or consequences) separately, without aggregating them into a single metric. This approach provides a detailed breakdown of different impacts, allowing decision-makers to weigh each outcome according to their own priorities and preferences.

### 2.3 Information sources

Eligible studies were identified from the following databases: PubMed, Embase, the Cochrane Library, and Web of Science from data inception to April 1, 2024. The databases were searched using key words and Medical Subject Headings (ie, MeSH). Only English-language publications were considered. We manually checked references in the included studies.

The search strategies for the above four databases were developed by the reviewer (A.L.) and reviewed by other reviewers (P.H. and Z.Y.). The full search strategies are detailed in Supplementary File S1.

### 2.4 Study selection

Two reviewers (A.L. and P.H.) independently screened titles and abstracts against the eligibility criteria. They then obtained the full texts of all potentially relevant publications and reviewed them to assess their compliance with the inclusion criteria. Any discrepancies were resolved through discussion.

### 2.5 Quality assessment

The quality of the included studies was evaluated using the Pediatric Quality Appraisal Questionnaire (PQAQ), a comprehensive tool validated for face and content validity, with strong interrater and test–retest reliability for assessing pediatric economic evaluations (23). The PQAQ comprises 57 items across 14 domains: (1) Economic evaluation, (2) Comparators, (3) Target population, (4) Time horizon, (5) Perspective, (6) Costs and resource use, (7) Outcomes, (8) Quality of life, (9) Analysis, (10) Discounting, (11) Incremental analysis, (12) Sensitivity analysis, (13) Conflict of interest, and (14) Conclusions. Of these, 46 items contribute to the study quality rating, with individual item scores assigned between 0 and 1. Based on the PQAQ score, studies were classified into three quality categories with a maximum score of 46: high (>75%), moderate (50%–74%), and low (<50%), as adopted by Sebastian et al. (24). Two authors conducted quality assessments independently, resolving any disagreements through discussion or by consulting a third author.

### 2.6 Data extraction

For each eligible study, two reviewers (A.L. and P.H.) independently extracted data by manually reviewing the included articles using a data extraction form. Z.Y. reviewed the data collected by the two reviewers to rule out any human error. In case of disagreement, a third author (Z.Y.) mediated discussions to reach a consensus. A list of all data entries collected is detailed in Supplementary File S2. Significant heterogeneity was observed among the included studies with respect to interventions, control groups, data sources, types of economic evaluations, study perspectives, price year, and currency. Consequently, a quantitative synthesis was not carried out.

## 3 Results

### 3.1 Characteristics of included studies

**Figure 1:**
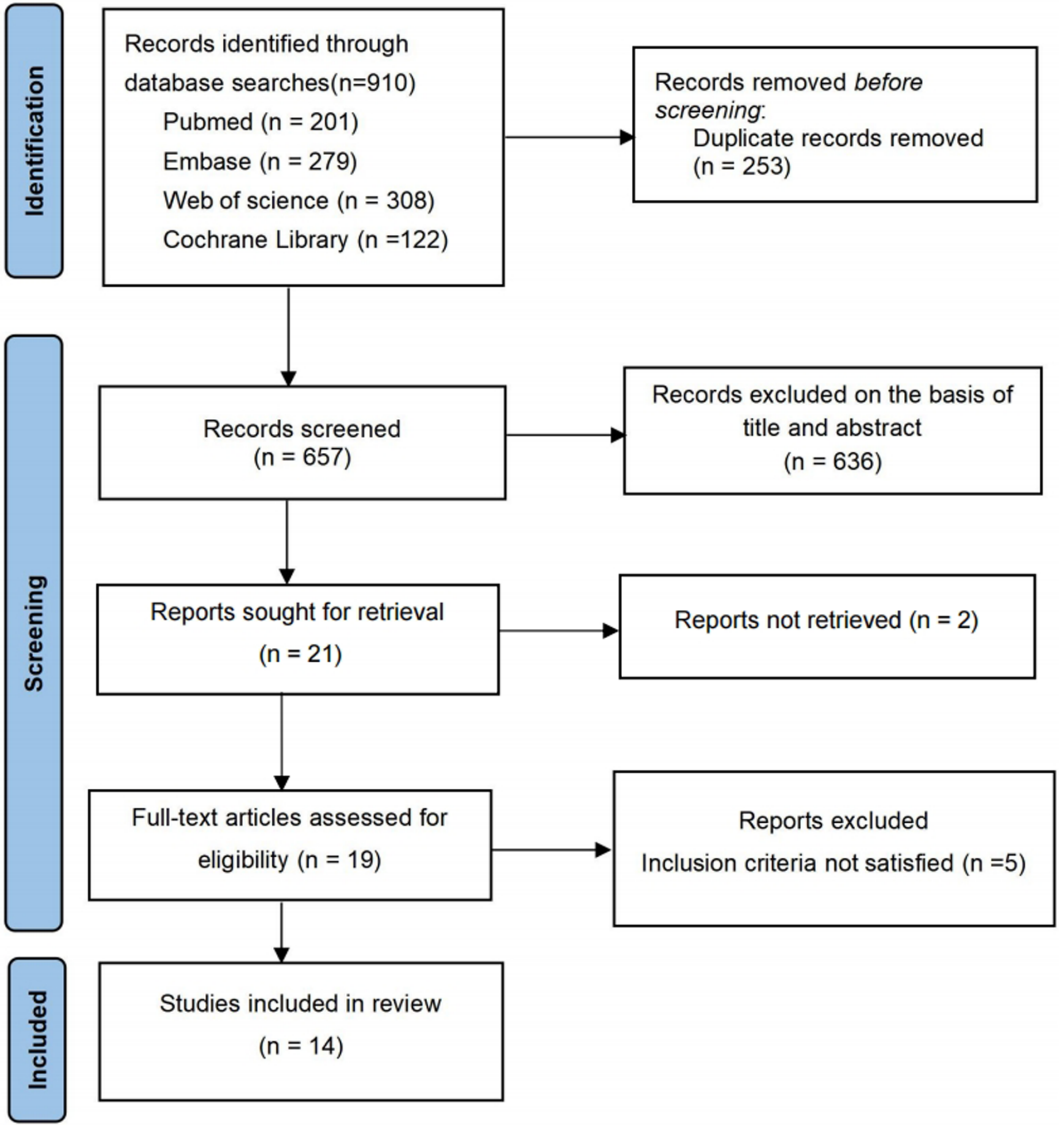
PRISMA flowchart shows the systematic search of the literature.

A total of 910 citations were identified through primary literature searches (Supplementary File S1). After removing 253 duplicates, 657 unique citations remained for further screening. Ultimately, 14 studies met the criteria for inclusion in the systematic review. The PRISMA flowchart illustrating the search and screening process is presented in Figure 1, while Table 1 provides a summary of the included studies along with their baseline characteristics (15, 16, 19–21, 25–33). Excluded studies and the reasons for their exclusion are shown in Supplementary File S3. The reviewed studies covered the period from 2012 to 2024 (Table 1). These studies were predominantly conducted in three countries worldwide, with the United States serving as the primary study site, where 85.7% (12/14) of the studies were conducted (Table 1). In terms of study setting, all took place in NICU settings. More than half of the economic evaluations (*n* = 10) were based on observational studies, whereas four studies (27, 28, 32, 33) employed model-based evaluations. The number of participants in each study varied substantially. For observational studies, this ranged from 64 (25) to 430 (19), while model-based studies included between 207 (32) and 1,000 participants (28).

**Table 1:** Summary of general characteristics of the studies.

Authors, year	Country	Setting	Data source	Study population	Subgroups	Intervention	Comparator
Tetarbe et al. (2024) (25)	USA	Level III NICU	Single center retrospective observational study	64 VLBW infants in the pre-EHM period and 57 VLBW infants in the post-EHM period	None	DHM and/or MOM fortified with HMDF	DHM and/or MOM fortified with MDF
Johnson et al. (2022) (19)	USA	Level III NICU	Single center prospective observational cohort from hospital discharges	430 VLBW infants	No complications,1complication,2 complication,3 complication	MOM feedings	Formula
Hanford et al. (2021) (26)	USA	Level III NICU	Single center retrospective cohort study	53 infants born <30 weeks or birth weights <1,100 g who received an EHD and 36 similar infants who received a BSD (MOM with bovine fortifier or preterm formula)	None	Exclusive human milk diet	MOM with bovine-based fortifier or preterm infant formula
Johnson et al. (2020) (21)	USA	Level III NICU	Single center retrospective cohort study	319 VLBW infants	None	MOM + donor milk	MOM + formula
Scholz et al. (2019) (27)	Germany	Germany	A decision tree model	The model population consists of the average, yearly number of VLBW newborns in Germany from the years 2012 to 2016	NEC, sepsis, NEC + sepsis and no complication	exclusive human milk (EHM) diet	Cow's milk-based fortifiers
Hampson et al. (2019) (28)	USA	tertiary NICU	Data from published studies (RCT and cohort)	A hypothetical population of 1,000 VLBW babies, all of whom are assumed to be admitted to a NICU	Medical NEC and surgical NEC	MOM supplemented with a human milk based fortifier	MOM supplemented with a cow's milk-based fortifier
Trang et al. (2018) (29)	Canada	tertiary NICU	Double-blinded RCT	363 VLBW infants <1,500 g	None	DHM	Bovine-based PTF
Patel et al. (2017) (15)	USA	Level III NICU	Single center prospective observational cohort study	254 VLBW infants with mean birth weight 1,027 ± 257 g and gestational age 27.8 ± 2.5 weeks	BPD, No BPD	MOM fortified with bovine human milk fortifier	Formula milk
Assad et al. (2016) (30)	USA	Level III community NICU	Single center retrospective chart review	293 preterm infants between gestational ages 23 to 34 weeks and birth weights between 490 and 1700g	None	EHM diet using either MOM or DHM and DHM-derived fortifier	Bovine-based fortifier and maternal milk; mixed combination of maternal milk, bovine-based fortifier and formula; and formula
Johnson et al. (2015) (20)	USA	Level III NICU	Single center prospective observational cohort study	291 VLBW infants	No NEC, NEC	MOM fortified with bovine human milk fortifier	Formula milk
Patel et al. (2013) (16)	USA	Level III NICU	Single center prospective observational cohort study	175 VLBW infants	No late-onset sepsis, Late-onset sepsis	MOM fortified with bovine human milk fortifier	Formula milk
Parker et al. (2012) (31)	USA	Level III NICU	Single center retrospective chart review	80 infants weighing less than 1,500 g, born prior to 32 weeks’ gestation	None	> 49% Breast Milk	100% Formula
Ganapathy et al. (2012) (32)	USA	Level III NICU	RCT and data from hospital discharges	207 VLBW infants (RCT), 2,560 EP infants in the final analytic sample derived from data	No NEC, medical NEC, and surgical NEC	Human milk-based diet composed of mother's milk fortified with a donor human milk-based HMF	Mother's milk fortified with a bovine milk-based HMF
Colaizy et al. (2016) (33)	USA	Level III NICU	Multiple center prospective cohort study (Glutamine trial)	848 ELBW infants	None	≥ 98% MOM fortified with bovine-based fortifier	exclusive preterm formula, a mixed diet (<98% MOM + preterm formula)

CMDF, cow's milk–derived fortiﬁers; DHM, donor human milk; EHM, exclusive human milk; HMDF, human milk–derived fortifiers; MOM, mother's own milk; NEC, necrotizing enterocolitis; NICU, neonatal intensive care unit; PTF, preterm formula; VLBW, very low birth weight.

### 3.2 Quality assessment of included studies

One study (29) was evaluated as high quality, while the remaining studies (15, 16, 19–21, 25–28, 30–33) were classified as moderate quality. Further details are provided in Supplementary File S4.

### 3.3 Analysis of clinical outcomes

1. NEC: Seven studies (21, 25, 26, 29–31, 33) successfully reported the incidence of NEC in both the intervention and control groups (Table 3). Four studies (21, 29, 30, 33) found a significantly lower incidence of NEC in the intervention group compared to the control group. Johnson et al. (21) reported a significantly lower incidence of NEC during the era of mother's own milk plus donor milk compared to the era of mother's own milk plus formula (1.8% vs. 6.0%, *P* = 0.048), with fewer infants requiring surgical treatment for NEC in the former. Similarly, Trang et al. (29) observed incidence of NEC stage ≥ I was significantly lower in the DHM group (3.9%) compared to the preterm formula (PTF) group (11.0%). Assad et al. (30) found a lower incidence of NEC in infants fed an exclusively human milk (EHM) diet compared to other groups. Colaizy et al. (33) reported an increased risk of NEC associated with exclusive preterm formula (aOR = 12.1, 95% CI 1.5, 94.2), or a mixed diet (aOR 8.7, 95% CI 1.2–65.2).
2. LOS: Seven studies (16, 21, 25, 26, 29–31) successfully reported the incidence of LOS in both the intervention and control groups (Table 3). Two studies (16, 26) reported a significantly lower incidence of LOS in the intervention group compared to the control group. Hanford et al. (26) found that infants fed an exclusive human diet (EHD) experienced a significant reduction in the rate of late-onset sepsis [7.55% in EHD to 22.22% in the bovine-based standard diet (BSD) group, *p* = 0.023]. Moreover, an EHD significantly reduced the odds of late-onset sepsis [adjusted odds ratio = 0.323; 95% CI (0.123, 0.768); *p* = 0.014]. Assad et al. (16) revealed that the increasing the average daily dose of HM (ADDHM) for the first 28 days post birth (ADDHM - Days 1–28) was associated with lower odds of sepsis [odds ratio 0.981, 95% CI (0.967–0.995), *P* = 0.008].
3. BPD: Five studies (15, 21, 25, 26, 30) documented the incidence of BPD in both the intervention and control groups (Table 3). Among these, one study (15) demonstrated a significantly lower incidence of BPD in the intervention group compared to the control. Patel et al. (15) found a 9.5% reduction in the odds of BPD for every 10% increase in the dose of MOM.
4. ROP: Five studies (21, 25, 26, 29, 30) documented the incidence of ROP in both the intervention and control groups (Table 3). Among these, one study demonstrated a significantly lower incidence of ROP in the intervention group compared to the control group. Hanford et al. (26) found that infants fed an EHD had a significantly reduced rate of severe ROP.
5. NICU length of stay: Eight studies (20, 21, 25, 26, 29–32) have examined the influence of human milk on the duration of hospitalization (Table 3). Of these, five studies (20, 21, 25, 30, 32) suggest that human milk can reduce the length of hospital stay, with reductions ranging from 3.9 days (32) to 6.3 days (25). Nevertheless, three studies (26, 29, 31) found no statistically significant difference in the length of hospital stay between the two groups.

### 3.4 Economics evaluation methods

1. Study Design: Six studies (15, 28, 30–33) utilized cost-consequence analysis (CCA), and five studies (19–21, 26, 29) applied cost-effectiveness analysis (CEA), while two studies (16, 25) utilized cost-benefit analysis (CBA). One study (27) utilized cost-utility analysis (CUA). With respect to modelling, one study (32) used a single cost-effectiveness model (assumed to be a decision model); two studies (27, 28) employed a decision tree model; and the fourth used Monte Carlo simulation (33). Studies by Scholz et al. (27), Hampson et al. (28), and Colaizy et al. (33) justified their model choice. The remaining model-based study did not provide any justification for its model choice (32) (Table 2).
2. Analytical Perspectives: All studies clearly reported their study perspective. Eleven studies (15, 16, 19–21, 25, 26, 30–33) evaluated costs solely from the healthcare system perspective. One study evaluated costs solely from a societal perspective (28). Scholz and Greiner (27) evaluated costs from both third-party payer and societal perspectives, while Trang et al. (29) analyzed costs from both the healthcare system and societal perspectives (Table 2).
3. Time Horizon: Among these, eleven studies (15, 16, 19–21, 26, 28, 30–33) adopted a short-term time horizon for evaluating costs and outcomes, extending until neonatal discharge from the hospital. One study (25) spanned a two-year period, one (27) followed a lifelong approach, tracking all costs and outcomes until the death of the cohort entering the model. In addition, Trang et al. (29) focused on the period from birth to 18 months of corrected age. None of these studies justified their choice of time horizon. Thirteen (93%) studies (15, 16, 19–21, 25–27, 29–33) stated the enrolment time frame for the infants (Table 2).
4. Price year/currency: Twelve (15, 16, 19–21, 25–29, 32, 33) (86%) studies specified their price year, while two studies (14%) (30, 31) did not specify a price year. All studies reported their currency.
5. Discount Rate: Twelve (86%) studies did not state a discount rate (15, 16, 19–21, 25, 26, 29–33), whereas two (27, 28) reported using an annual discount rate of 3%, for both costs and benefits as recommended by the US Second Panel on Cost-Effectiveness in Health and Medicine (34) (Table 2).
6. Resource use and costs: The choice of inclusion of a particular type of resource use and cost varied according to the study purpose, perspective, time horizon and the nature of the intervention/comparator being evaluated. Costs tended to be categorized into direct medical care costs [e.g., NICU, NEC treatment (medical and surgical), sepsis and hospitalization costs] (28–30, 32); informal and non-medical care costs (e.g., caregiver transportation and labor market earnings lost) (29, 33); indirect costs incurred by institutions (e.g., administration, human resources and plant operations) (29); societal costs (27–29); enteral feeding costs (29); parenteral feeding costs (25, 26); and resource use and costs of the DHM/other diet provision such as formula milk (detailed information is presented in Table 2) (26, 28, 30, 32).
7. Sensitivity Analysis: Sensitivity analyses were performed in six papers (21, 26–29, 32). Two studies (27, 29) employed deterministic sensitivity analyses, while one study (32) applied one-way and two-way parameter percentage changes to construct an expected cost calculator. Further details are provided in Table 3.
8. Narrative synthesis of economic evaluations: We cannot compare results of economic evaluations that assess health care interventions, which have been conducted in different regions/settings and times. This is due to notable differences in the funding of health care systems, the treatments and care pathways, and baseline population and demographic characteristics around the world. Despite the heterogenous methods of economic evaluations used prohibiting direct comparison between studies, all human milk interventions indicated cost-effective or cost saving results.

Four studies (20, 21, 25, 28) reported cost savings in NICU hospitalizations. Tetarbe et al. (25) reported that EHM feeding led to a cost saving of $1,813,444. Johnson et al. (21) reported that MOM combined with donor milk was associated with $15,555 lower costs per infant (*P* = 0.045) and saved $1,812 per percentage point decrease in NEC incidence. Hampson et al. (28) demonstrated that EHM diet generated substantial cost savings of $16,309 per infant by reducing adverse clinical events. Johnson et al. (20) indicated that each additional ml (kg. day) of HM during Days 1–14 decreased non-NEC-related NICU costs by $534 (*p* < 0.001).

**Table 2:** Detailed account of the economic evaluation methods — part 1.

Authors, year	Type of economic evaluation	Model type	Study perspective	Time horizon	Price year/currency	Discount rate	Resource use and costs	Detail resource use and costs (MOM/DHM/other diet provision)
Tetarbe et al. (2024) (25)	CBA	NR	HCS	2 years	2020–2021/USD	NR	Hospitalization costs: stay for VLBW infants/medical NEC/late-onset sepsis/BPD/ROP/total parenteral nutrition costs	DHM: $27 to $590 per infant
Johnson et al. (2022) (19)	CEA	NR	HCS	NICU hospitalization	2016/USD	NR	Hospitalization costs: initial stay for VLBW infants/NEC/late-onset sepsis/BPD costs	MOM: $538per infant; Formula: $302 per infant
Hanford et al. (2021) (26)	CEA	NR	HCS	NICU hospitalization	2016/USD	NR	Hospitalization costs: initial stay for VLBW infants/NEC (medical and surgical)/late-onset sepsis/BPD/severe ROP/total parenteral nutrition costs	Cost of human donor milk for 36 infants in 2016 was $365,231.
Johnson et al. (2020) (21)	CEA	NR	HCS	NICU hospitalization	2016/USD	NR	Hospitalization costs: stay for VLBW infants/NEC/late-onset sepsis/BPD ROP/severe brain injury costs	Formula: $3.30 per 100 ml; MOM: $12.35 per 100 ml; donor milk: $21.18 per 100 ml
Scholz et al. (2019) (27)	CUA	decision tree model	TPP, SP	life-long	2017/EUR	3%	Hospitalization costs: initial stay for VLBW infants/NEC (medical and surgical)/sepsis costs, sensitivity analysis: societal costs	Fortifier: €6 per ml; donor milk: €65 per liter.
Hampson et al. (2019) (28)	CCA	decision tree model	SP	NICU hospitalization	2016/USD	3% for both costs and benefits	Hospitalization costs: initial stay for VLBW infants/NEC (medical and surgical) late-onset sepsis costs, sensitivity analysis: societal costs	30 ml Pro lact + 6 product: $187.50, DHM: $183; total EHM diet cost: $7,731; cow's milk: $226
Trang et al. (2018) (29)	CEA	NR	SP, HCS	birth to 18 m CA	2015/CAD	NR	readmissions costs: physician fees; enteral feeds, indirect, informal non-medical costs, societal costs	DHM unit cost: 4.95 (3–7.6) Canadian $/ounce; bovine-based PTF: 0.13 Canadian $/ounce; fortifier: 0.14 Canadian $/ounce
Patel et al. (2017) (15)	CCA	NR	HCS	NICU hospitalization	2014/USD	NR	Hospitalization costs: initial stay for VLBW infants/BPD costs	NR
Assad et al. (2016) (30)	CCA	NR	HCS	NICU hospitalization	NR/USD	NR	Hospitalization costs: length of stay for VLBW infants, physician charges	EHM group: donor milk and donor milk-derived fortifier costs ($125 – $250/100 ml bottle)
Johnson et al. (2015) (20)	CEA	NR	HCS	NICU hospitalization	2012/USD	NR	Hospitalization costs: initial stay for VLBW infants/NEC costs	NR
Patel et al. (2013) (16)	CBA	NR	HCS	NICU hospitalization	2010/USD	NR	Hospitalization costs: initial stay for VLBW infants/late-onset sepsis costs	NR
Parker et al. (2012) (31)	CCA	NR	HCS	NICU hospitalization	NR/USD	NR	Hospitalization costs: initial stay for VLBW infants/NEC/late-onset sepsis costs	The potential expense of providing breast milk to VLBW infants was not taken into account. Formula was provided by formula companies free of charge
Ganapathy et al. (2012) (32)	CCA	Assumed a decision model	HCS	NICU hospitalization	2011/USD	NR	NEC (medical and surgical) hospitalization costs, net savings in hospital costs	Pro lact/H2MF: $6.25/ml, DHM: $3.00/ounce ($0.10/ml); bovine milk-based HMF: $1.30/packet, PTF: $1.00/ounce ($0.03/ml)
Colaizy et al. (2016) (33)	CCA	Monte Carlo simulation	HCS	NICU hospitalization	2014/USD	NR	NEC (medical and surgical) direct hospital costs and indirect non-medical costs	≥ 98% MOM fortified with bovine-based fortifier: $34 and $172 per infant; formula: $213 per infant; donor human milk: $1,005 per infant

BPD, bronchopulmonary dysplasia; CA, corrected age; CAD, Canadian dollars; CBA, cost-benefit analyses; CCA, cost-consequence analysis; CEA, cost-effectiveness analyses; CUA, cost-utility analyses; DHM, donor human milk; EHM, exclusive human milk; HCS, healthcare system; HMD, exclusive human milk diet; HMF, human milk fortifier; MOM, mother's own milk; NEC, necrotizing enterocolitis; NICU, neonatal intensive care unit; NR, not reported; PTF, preterm formula; ROP, retinopathy of prematurity; SP, societal perspective; TPP, third-party payer; USD, US dollar; VLBW, very low birth weight.

**Table 3:** Detailed account of the economic evaluation methods — part 2.

Authors, year	Type of sensitivity analysis	Clinical outcomes	Benefit of health	Economic outcomes	Conclusion
Tetarbe et al. (2024) (25)	NR	NEC (medical and surgical), late onset sepsis, BPD, severe ROP, Days on parenteral nutrition, length of stay	The EHM feeding guideline led to a reduction in the mean length of stay and mean days of PN use by 6.3 and 6.8 days per infant, respectively. No significant difference in incidence of short-term morbidities was observed.	Savings from reduced TPN days and length of stay was estimated to be $1,813,444 ($31,815 per infant).	Implementation of EHM-based feeding in VLBW infants is a cost-effective option for neonatal intensive care units that can result in reduced length of stay and days on PN without adversely impacting short-term morbidities.
Johnson et al. (2022) (19)	NR	NEC (medical and radiologic), late onset sepsis, BPD, length of stay	There were significant differences in infant characteristics by the number of complications, including MOM feedings, NICU length of stay, and NICU hospitalization costs.	The mean NICU hospitalization cost was $190,586 (standard deviation $119,235). The marginal cost of sepsis was $27,890 (95% CI $2,934–$52,646), of NEC was $46,103 (95% CI $16,829–$75,377), and of BPD was $41,976 (95% CI $24,660–59,292). The cumulative proportion of MOM during the NICU hospitalization was not significantly associated with cost.	A reduction in the incidence of complications that are potentially preventable with MOM intake has significant cost implications. Hospitals should prioritize investments in initiatives to support MOM feedings in the NICU.
Hanford et al. (2021) (26)	Inflation adjustments	NEC (medical and surgical), late onset sepsis, BPD, severe ROP, Days on parenteral nutrition, length of stay, deaths	An EHD significantly decreased the odds of severe ROP and LOS	Analysis of cost-effectiveness of an EHD relative to a BSD based on the incremental costs of these co-morbidities determined the net loss in direct hospital costs per patient were estimated to be $420 in 2016 US dollars	This study found that an EHD signiﬁcantly decreased the odds of severe ROP and late onset sepsis; though not signiﬁcant, there was a positive trend in decreasing cases of medical NEC; our surgical NEC rates dropped to 0. The beneﬁts of human milk are vital, and the costs are nominal.
Johnson et al. (2020) (21)	Bootstrapping	NEC, late onset sepsis, BPD, ROP (stage 3 or greater), Severe brain injury, Days on parenteral nutrition, length of stay	Infants receiving MOM + donor milk had a lower incidence of NEC than infants receiving MOM + formula (1.8% vs. 6.0%, *P* = .048).	Total hospital + feeding median costs were $169 555 for MOM + donor milk and $185 740 for MOM + formula, with median feeding costs of $1317 and $936, respectively. MOM + donor milk was associated with $15 555 lower costs per infant and saved $1,812 per percentage point decrease in NEC incidence.	The additional cost of a donor milk program was small compared with the cost of a NICU hospitalization. After its introduction, the NEC incidence was signiﬁcantly lower with small cost savings per case. We speculate that NICUs with greater NEC rates may have greater cost savings.
Scholz et al. (2019) (27)	Deterministic sensitivity analyses, probabilistic sensitivity analyses	NEC (medical and surgical), late onset sepsis, BPD, severe ROP	The EHM strategy can be considered a cost-effective new treatment strategy for very low birth weight newborns in Germany from a TPP perspective under a maximal WTP threshold of€45,790/LYG. Only decreasing the effectiveness against more than one complication concurrently makes the ICER increase above the WTP threshold of€45,790/LYG recommended by WHO for Germany.	In the base case, the EHM was estimated to be cost-effective compared to the current nutrition for VLBW with an ICER of €28,325 per LYG. From a societal perspective, the ICER is €27,494/LYG using a friction cost approach and €16,112/LYG using a human capital approach.	Adopting EHM as the standard approach to nutrition is a cost-effective intervention for VLBW infants in Germany.
Hampson et al. (2019) (28)	(1) Various threshold analyses to explore incidence rates of late onset sepsis/NEC: EHM to be cost saving. (2) Lower/higher cost scenarios. (3) Some examples of wider societal costs. (4) Case where mortality for usual care group was estimated from retrospective cohort study, with treatment effect of EHM on mortality taken from trial	deaths (initial hospital stay), NEC (medical and surgical), late onset sepsis and other infections	EHM substantially reduces mortality and improves other health outcomes	EHM generates substantial cost savings of $16,309 per infant by reducing adverse clinical events. Cost savings increase to $117, 239 per infant when wider societal costs are included.	EHM is dominant in cost-effectiveness terms that it is both cost savings and clinically beneficial, for VLBW babies in a US-based setting. These findings indicate that the use of EHM rather than usual care in a US setting would reduce costs for health care payer and lead to improved health outcomes for VLBW babies.
Trang et al. (2018) (29)	Deterministic sensitivity analyses	NEC, late-onset sepsis, severe ROP, length of stay	There were no differences in major clinical outcomes during initial hospitalization except for the incidence of NEC.	Examination of post discharge to 18 months’ CA costs revealed lower costs for infants randomly assigned to the DHM versus PTF group. Post discharge, caregivers of infants randomly assigned to the DHM group had significantly lower productivity losses than infants randomly assigned to the PTF group.	In a high mother's milk use setting, total costs from a societal perspective to 18 months of providing supplemental DHM versus PTF to VLBW infants did not differ, although post discharge costs were lower in DHM group. Although supplemental DHM was not cost saving, it reduced NEC supporting its use over PTF
Patel et al. (2017) (15)	NR	BPD	a 9.5% reduction in the odds of BPD for every 10% increase in OMM dose.	After controlling for demographic and clinical factors, BPD was associated with an increase of US $41 929 in NICU costs.	Increased dose of OMM feedings from birth to 36 weeks PMA was associated with a reduction in the odds of BPD in VLBW infants. Thus, high-dose MOM feeding may be an inexpensive, effective strategy to help reduce the risk of this costly multifactorial morbidity.
Assad et al. (2016) (30)	NR	hospital stays, NEC/intolerance incidence, weight gain, time to full feed, BPD, ROP, sepsis	Feeding intolerance occurred less often, number of days to full feeds was lower, incidence of NEC was lower in those fed an EHM diet compared with the other groups.	total hospitalization costs were lower by up to $106,968 per infant in those fed an EHM diet compared with the other groups	Implementing EHM diet in VLBW infants has led to a significant decrease in incidence of NEC. Other benefits of this diet include decreased feeding intolerance, shorter time to full feeds, shorter length of stay, and lower hospital/physician charges for EP and VLBW infants.
Johnson et al. (2015) (20)	NR	NEC, length of stay	NR	NEC was associated with a marginal increase in costs of $43,818, after controlling for demographic characteristics, risk of NEC and average daily dose of HM during Days 1–14. Each additional ml/kg/day of HM during Days 1–14 decreased non-NEC-related NICU costs by $534	Avoidance of formula and use of exclusive HM feedings during the first 14 days of life is an effective strategy to reduce the risk of NEC and resulting NICU costs in VLBW infants. Hospitals investing in initiatives to feed exclusive HM during the first 14 days of life could substantially reduce NEC-related NICU hospitalization costs.
Patel et al. (2013) (16)	NR	late onset sepsis	increasing ADDHM - Days 1–28 was associated with lower odds of sepsis	NICU costs were lowest in the VLBW infants who received the highest ADDHM-Days 1–28.	A dose–response relationship was demonstrated between ADDHM-Days 1–28 and a reduction in the odds of sepsis and associated NICU costs after controlling for propensity score. For every HM dose increase of 10 ml/(kg. day), the odds of sepsis decreased by 19%. NICU costs were lowest in the VLBW infants who received the highest ADDHM-Days 1–28.
Parker et al. (2012) (31)	NR	NEC, BPD, late-onset sepsis, length of stay	There were no statistically significant differences in incidence of NEC or LOS between groups.	No statistically significant differences in length of stay or cost of care were found between infants fed at least 50% breast milk and those who were exclusively formula fed.	This article presents a descriptive comparative study on the effect of providing at least 50% breast milk feedings compared with formula feeding on days to discharge and cost of hospitalization in VLBW infants in the NICU. It also provides information concerning cost of care and length of stay in VLBW and infants weighing less than 1,000 g.
Ganapathy et al. (2012) (32)	One-way/two-way percentage changes in parameters. Cost savings from donor HMF strategy were sensitive to price quantity of donor HMF, percentage reduction in risk of overall and surgical NEC achieved and incremental costs of surgical NEC	NEC, length of stay	Extremely premature infants fed with 100% human milk-based products had lower expected NICU length of stay	Extremely premature infants fed with 100% human milk-based products had lower total expected costs of hospitalization, resulting in net direct savings of 3.9 NICU days and $8,167.17 per extremely premature infant.	Compared with feeding EP infants with mother's milk fortified with bovine milk-based supplements, a 100% human milk-based diet that includes mother's milk fortified with donor human milk-based HMF may result in potential net savings on medical care resources by preventing NEC.
Colaizy et al. (2016) (33)	NR	NEC	In adjusted models, compared with infants fed predominantly MOM, we found an increased risk of NEC associated with exclusive preterm formula (a OR = 12.1, 95% CI 1.5, 94.2), or a mixed diet (a OR 8.7, 95% CI 1.2–65.2).	These models estimated an annual cost of suboptimal feeding of ELBW infants of $27.1 million (CI $24million, $30.4 million) in direct medical costs, $563,655 (CI $476,191, $599,069) in indirect nonmedical costs.	Among ELBW infants, not being fed predominantly MM is associated with an increased risk of NEC. Efforts to support milk production by mothers of ELBW infants may prevent infant deaths and reduce costs.

ADDHM, average daily dose of HM; BPD, bronchopulmonary dysplasia; BSD, bovine-based standard diet; CA, corrected age; DHM, donor human milk; EHD, exclusive human diet; EHM, exclusive human milk; ICER, incremental cost-effectiveness ratio; LYG, Life-Year-Gained; MOM, mother's own milk; NEC, necrotizing enterocolitis; NICU, neonatal intensive care unit; NR, not reported; OMM, own mother's milk; PN, parenteral nutrition; PTF, preterm formula; ROP, retinopathy of prematurity; TPN, total parenteral nutrition; VLBW, very low birth weight.

Two studies provided marginal costs (19, 20). Johnson et al. (19) reported that the marginal cost of sepsis was $27,890 (95% CI $2,934–$52,646), of NEC was $46,103 (95% CI $16,829–$75,377), and of BPD was $41,976 (95% CI $24,660–59,292). Johnson et al. (20) reported that NEC was associated with a marginal increase in costs of $43,818.

Four studies provided incremental costs (15, 16, 21, 32). Johnson et al. (21) found that NEC was associated with $66,015 greater costs per infant (*P* < 0.001), and BPD was associated with $74,084 greater costs per infant (*P* < 0.001). Patel et al. (15) indicated that BPD was associated with an increase of US $41,929 in NICU costs. Patel et al. (16) reported that increasing ADDHM -Days 1–28 was associated with significantly lower NICU costs. Average costs were $31 514 lower for infants with ADDHM-Days 1–28 ≥ 50 ml (kg. day) and $20 384 lower for infants with ADDHM-Days 1–28 25–49.99 ml (kg. day), when compared with infants with ADDHM-Days 1–28 less than 25 ml (kg. day). Ganapathy et al. (32) indicated that the adjusted incremental costs of medical NEC and surgical NEC, over and above the average costs incurred for extremely premature infants without NEC, in 2011 US$, were $74,004 (95%CI, $47,051-$100,957) and $198,040 (95%CI, $159,261-$236,819) per infant, respectively.

Two study offered ICER (27, 29). Scholz et al. (27) found that in the base case, the EHM diet was estimated to be cost-effective compared to the current nutrition for VLBW newborns, with an incremental ICER of €28,325 per Life-Year-Gained. Trang et al. (33) indicated DHM cost an additional $5,328 per case of averted NEC (ICER: $5,328 per case of averted NEC).

9. Generalizability: Four (29%) of the studies reported information regarding the generalizability of their results (15, 16, 28, 29). These studies presented differing perspectives on the generalizability of their findings. Hampson et al. (28) indicated that since the clinical and resource use data are specific to the United States, no strong conclusions on the applicability of their findings to other contexts can be made. However, the cost-saving potential identified in their analysis suggests that further investigation into the cost-effectiveness of an EHM diet in different settings may be warranted. Trang et al. (29) noted that their study, conducted in a single Canadian urban area, may have limited generalizability due to potential variations in maternal milk feeding practices or associated costs in other regions. Similarly, Patel et al. (15) highlighted that their data from a single institution may restrict generalizability, and Patel et al. (16) acknowledged that their single-center study also potentially limits the broader applicability of their findings.

## 4 Discussion

This systematic review is the first to explore the economic evaluations of human milk for VPIs. Fourteen studies from three high-income countries were included. We found that, among the economic evaluations, all HM interventions demonstrated cost-effective or cost saving outcomes. Research indicates a dose-response relationship between HM consumption and the reduction of morbidities, with higher doses leading to greater risk reduction (13, 15, 16). Critical periods during NICU hospitalization, such as the first 10 or 28 days of life, are vital for VPIs to receive high doses of HM (13, 16, 35). Xu et al. found that a daily threshold of ≥50 ml (kg. day) of HM in the first 4 weeks of life was linked to lower incidence of various complications in very low birth weight (VLBW) infants, including BPD, NEC, LOS, and extrauterine growth restriction (13). HM is recommended as the primary nutritional source for NICU patients, particularly VLBW infants at high risk for complications (36). Promoting breastfeeding in critical care settings is associated with greater health benefits and lower costs compared to preterm infant formula, suggesting potential cost-effectiveness (37).

Johnson et al. (38) highlighted the economic benefits of HM feeding during NICU hospitalization; however, they did not perform a systematic evaluation. Buckle et al. (39) reviewed the cost and cost-effectiveness of donor human milk (DHM) specifically for the prevention of necrotizing enterocolitis (NEC), concentrating primarily on this single outcome. Their findings suggested that DHM use is likely cost-effective; nonetheless, they recommended that comprehensive economic evaluations comparing DHM with standard feeding protocols in infants are necessary to strengthen the evidence base. Similarly, Zanganeh et al. (40) conducted a systematic review of economic evaluations regarding DHM vs. standard feeding in infants, focusing exclusively on DHM. They reported that DHM interventions consistently yielded cost-effective or cost-saving results and proposed that future studies should provide more detailed insights into the long-term costs and outcomes associated with DHM. In contrast, our review examines the association between HM feeding and very preterm infants from both clinical and health economics perspectives.

### 4.1 The relation between HM with prematurity-related morbidities

NEC is a severe, inflammation-related morbidity affecting approximately 7% of VLBW infants (41). NEC not only prolongs NICU hospitalization but also raises daily NICU costs due to the utilization of expensive pharmaceutical products, therapies, surgeries, and other services, resulting in an additional cost of $66,015 per infant (21). The incremental cost of NEC varies from $43,818 (20) to $46,103 (19) per infant and $223 per day (95% CI: $100–$346) (19). Evidence indicates that HM may be cost-saving or cost-effective in the context of NEC among preterm infants, although the economic impact may differ across settings (21, 29, 30). The use of donor milk was associated with a saving of $1,812 per percentage point reduction in NEC incidence (21), while DHM incurred an additional cost of $5,328 per averted NEC case (29).

NICU hospitalization costs attributable to LOS range from $17,822 to $27,890 (in 2016 US$) (19, 26). One study demonstrated a strong causal relationship between LOS and MOM feedings (19). Higher doses and longer durations of MOM during NICU hospitalization are associated with reduced risks of sepsis (16) and significant cost savings with institutional investments in MOM feedings (19). A 19% reduction in the odds of developing sepsis is observed for every 10 ml (kg. day) increase in the average daily dose of HM during Days 1–28 (16).

Both HM and DHM significantly decrease the incidence of BPD compared to preterm formula (30, 42). Even when the amount of HM is insufficient, feeding more than 50% of the total volume still offers protective effects against BPD. There is a dose-dependent relationship between MOM consumed in the NICU and BPD occurrence, influencing associated healthcare costs (13). For every 10% increase in the proportion of MOM, the odds of BPD were reduced by 9.5% (15).

The incremental cost associated with severe ROP was $39,344 for an infant born at 27 weeks' gestational age (26). After adjusting for sex, race, gestational age, and birth weight, infants who received EHD demonstrated a significant 65.1% decrease in the odds of severe ROP compared to those fed a bovine-based standard diet (30).

Our study focused on research that addressed both preterm-related complications and health economics, excluding studies that solely examined the relationship between HM and preterm-related complications. Limited data is available on the positive effects of HM feeding on these conditions.

### 4.2 The relation between HM with NICU hospitalization costs

NICU hospitalization costs include physician costs, nursing costs, respiratory costs, transfusion costs, and procedure costs, among others (3). Rios et al. (3) obtained patient resource use data from the Canadian Neonatal Network database. Cost estimates were generated by matching patient resource use data to the appropriate unit costs. All cost estimates were calculated from the perspective of a provincial public payer. The median cost of NICU hospitalization was estimated at $30,572 ($16,597-$51,857) (in 2017 Canadian dollars) for infants with a gestational age of 29–32 weeks and $100,440 ($56,858-$159,386) (in 2017 Canadian dollars) for those with a gestational age of less than 29 weeks. Johnson et al. (19) reported that the mean NICU hospitalization cost of VPIs was $190,586 (in 2016 US$).

Most included studies suggest that human milk feedings can decrease NICU hospitalization costs for VPIs. HM feedings are cost-effective, although different studies have yielded varied conclusions regarding the incidence rates of various complications in preterm infants. HM feedings may also have a direct impact on healthcare costs independent of their association with morbidities such as LOS, NEC, BPD, and ROP. One study evaluated the direct relationship between the dose of HM and healthcare costs, after controlling for the presence of one or more morbidities (16). The study found significantly lower NICU hospitalization costs with higher doses of HM, defined as ml (kg. day), in the first 28 days post-birth.

To ensure an adequate milk supply, NICUs must bear the cost of acquiring HM. Johnson et al. (21) found that the mean cost per 100 ml was $3.30 for formula, $12.35 for MOM, and $21.18 for donor milk (in 2016 US dollars). However, the subsequent direct and indirect cost savings from HM feedings likely far outweigh the hospital's expenses for acquiring HM in most instances. Scholz et al. (27) used a decision tree model to calculate the cost-effectiveness of the EHM diet and found that adopting EHM diet as the standard approach to nutrition is a cost-effective intervention for VLBW newborns in Germany. Hampson et al. (28) conducted an economic analysis of EHM diet compared to cow's milk among VLBW babies in the US and found that an EHM diet is dominant in cost-effectiveness terms; it is both cost-saving and clinically beneficial for VLBW babies in a US-based setting.

### 4.3 Economics evaluation methods

The majority of the studies reported clinical outcome measures (e.g., incidence of NEC). Only one of the fourteen studies (27) reported health-related outcome measures (e.g., Life-Year-Gained) commonly used in economic evaluations. Six studies (15, 28, 30–33) applied a CCA approach with costs savings as an outcome; this approach is considered a type of cost-benefit analysis. Consideration of a broader range of outcomes beyond the health sector allows for inclusion of benefits and costs from multiple sectors.

Model-based evaluations offer the opportunity to improve the generalizability of findings and evaluate the longer-term costs and benefits of HM. These evaluations are critical as policy-making tools, often informing resource allocation decisions. One of the model-based studies provided data over a longer time horizon (27). However, one study did not explicitly mention procedures for model validation (32). Moreover, the clinical and resource utilization data are specific to a single country. Therefore, the applicability of findings to other settings, particularly from high-income to low-income countries, may be limited.

Many evaluations in this review lacked the application of a discount rate. The majority of studies utilized a short-term horizon to evaluate costs and outcomes, extending until neonatal discharge from the hospital, and thus may not accurately reflect longer term health effects or consider all aspects of economic evaluations. The methods for collecting resource utilization and types of costs included varied across studies. The majority of the studies did not report which cost components were excluded from their analyses. Future studies should clearly specify which costs are included and excluded. Four studies (27–29, 33) included informal and non-medical care costs, as well as indirect and societal costs. It is considered good practice to report findings both including and excluding informal and indirect costs. Incorporating these types of costs (e.g., costs incurred by families) may influence management recommendations. To determine the macroeconomic benefits of HM in reducing the incidence of NEC, BPD and LOS, an analysis of lifetime costs would be useful. However, a lifetime model comparing the economic impact of HM to formula feeding would require significant assumptions, potentially introducing high uncertainty. Establishing causality in this area is particularly challenging and requires substantial data, which may not be available.

Economic evaluations inherently contain some degree of uncertainty. To assess this uncertainty, various sensitivity analyses can be applied to evaluate how sensitive results are to uncertain parameters. The selection of sensitivity analysis method may depend on the methodology, type of economic evaluation (trial-based or model-based), or the intervention's setting. Notably, six studies (21, 26–29, 32) conducted sensitivity analyses to assess the robustness of their results.

While all HM interventions reviewed appear cost-effective or even cost-saving, variation exists based on intervention design. The narrative synthesis of economic evaluations, including appraisal of applied methods and assessment of study quality, provides valuable insights for health economists, modelers, and future research directions in this field.

### 4.4 Strengths

Our systematic review has several strengths. Firstly, it is the first comprehensive analysis of economic evaluations of HM for VPIs. Secondly, we employed a thorough search strategy that spanned multiple databases and included additional reference checks to encompass a wide range of relevant published studies. Thirdly, we strictly followed PRISMA guidelines for duplicate screening, data extraction, and quality assessment.

### 4.5 Limitations

Despite the use of scientific and systematic methods to minimize deviations, it is important to acknowledge several limitations in this study. Firstly, quantitative synthesis was not carried out due to significant heterogeneity across studies. While most studies focused on medical costs, some provided detailed cost breakdowns while others did not, limiting quantitative analysis and horizontal comparison. The definitions of complications associated with preterm infants are not standardized. For example, in the case of NEC, most studies (19, 21, 25) use Bell stage II or higher, some use medical and surgical classifications (26), and others use ICD codes (27). Most included studies do not provide detailed information on the dosage, proportion, and duration of maternal or donor breast milk, making cross-study comparisons infeasible. Therefore, a qualitative summary of evidence was conducted with cautious interpretation of outcomes. However, current published studies are informative and provide the basis for further research on economic evaluations of human milk for very preterm infants. Furthermore, all included studies only considered medical costs, neglecting maternal opportunity costs due to time spent pumping and other costs incurred by the mother. Future research should explore economic evaluations from a societal perspective. Additionally, it is important to note that the included studies were conducted in countries with ample medical resources, so generalizing these findings to countries with limited resources should be done cautiously, as costs and payer arrangements may vary across different economic levels.

## 5 Conclusions

This study represents the first systematic review on the economic evaluations of human milk for very preterm infants. This systematic review suggests that economic evaluation of HM for VPIs is an expanding area of research, and current economic evaluations are mainly set in developed countries. The findings suggest that human milk for very preterm infants yield significant economic benefits during critical periods of NICU hospitalization. Optimizing human milk feedings in the NICU emerges as a cost-effective strategy for enhancing infant health outcomes in this highly vulnerable patient population. There was considerable heterogeneity and deficiencies in the included studies. Standardized and high-quality studies are needed to determine the cost-effectiveness of human milk for very preterm infants in the future.

## Data Availability

The original contributions presented in the study are included in the article/Supplementary Material, further inquiries can be directed to the corresponding authors.
