# Supplementary material for: Economic evaluations of human milk for very preterm infants: a systematic review
Source: Front Pediatr. 2025 Mar 20;13:1534773. doi: 10.3389/fped.2025.1534773 (PMC11965692; doi:10.3389/fped.2025.1534773)
Supplement: Supplementary file 1 [file Datasheet1.docx]

Supplementary Material

**Contents of supplementary material**

1. Supplementary File S1. Search strategy for databases

2. Supplementary File S2. Data items extracted from studies

3. Supplementary File S3. List of references with final exclusion reasons

4. Supplementary File S4. Pediatric Quality Appraisal Questionnaire (PQAQ) of Economic Evaluations of Vision Screening Strategies in Children under the age of Six Years

5.Supplementary File S5. Completed PRISMA checklist

**Supplementary File S1. Search strategy for four databases**

| **PubMed** | | |
| --- | --- | --- |
| **Search number** | Query | Results |
| #1 | "Costs and Cost Analysis"[MeSH Terms] OR "Cost-Benefit Analysis"[MeSH Terms] OR "Cost of Illness"[MeSH Terms] OR "Cost-Effectiveness Analysis"[MeSH Terms] OR "Cost Savings"[MeSH Terms] | 274,024 |
| #2 | "economic evaluation"[Title/Abstract] OR "economic analys*"[Title/Abstract] OR "cost analys*"[Title/Abstract] OR "cost effective* analys*"[Title/Abstract] OR "cost effective* analys*"[Title/Abstract] OR "cost benefit* analys*"[Title/Abstract] OR "cost utility* analys*"[Title/Abstract] OR "cost benefit* analys*"[Title/Abstract] OR "cost utility* analys*"[Title/Abstract] OR "Cost"[Title/Abstract] OR "Cost Savings"[Title/Abstract] OR "Cost Analysis"[Title/Abstract] OR "cost-effectiveness"[Title/Abstract] OR "economic evaluation"[Title/Abstract] OR "cost-benefit"[Title/Abstract] OR "cost-utility analysis"[Title/Abstract] | 624,581 |
| #3 | #1 OR #2 | 766,137 |
| #4 | "infant, extremely premature"[MeSH Terms] OR "Premature Birth"[MeSH Terms] OR "infant, premature"[MeSH Terms] OR "Infant, Very Low Birth Weight"[MeSH Terms] OR "Infant, Extremely Low Birth Weight"[MeSH Terms] | 94,359 |
| #5 | "infant premature"[Title/Abstract] OR "infants premature"[Title/Abstract] OR "premature infant"[Title/Abstract] OR "preterm infants"[Title/Abstract] OR "infant preterm"[Title/Abstract] OR "infants preterm"[Title/Abstract] OR "preterm infant"[Title/Abstract] OR "premature infants"[Title/Abstract] OR "neonatal prematurity"[Title/Abstract] OR "prematurity neonatal"[Title/Abstract] OR "premature infant*"[Title/Abstract] OR "prematurity infant*"[Title/Abstract] OR "preterm infant*"[Title/Abstract] OR "low birth weight"[Title/Abstract] OR "low birthweight"[Title/Abstract] OR "VLBW"[Title/Abstract] OR "LBW"[Title/Abstract] | 94,061 |
| #6 | #4 OR #5 | 138,408 |
| #7 | "milk, human"[MeSH Terms] OR "Breast Milk Expression"[MeSH Terms] OR "Breast Feeding"[MeSH Terms] | 62,276 |
| #8 | "milk human"[Title/Abstract] OR "breast milk"[Title/Abstract] OR "milk breast"[Title/Abstract] OR "human milk"[Title/Abstract] OR "Breast Feeding"[Title/Abstract] | 41,766 |
| #9 | #7 OR #8 | 76,033 |
| #10 | #3 AND #6 AND #9 | 201 |

| **Cochrane library** | | |
| --- | --- | --- |
| **ID** | Search | Hits |
| #1 | (Costs and Cost Analysis):ti,ab,kw OR (Cost-Benefit Analysis):ti,ab,kw OR (Cost of Illness):ti,ab,kw OR (Cost-Effectiveness Analysis):ti,ab,kw OR (Cost Savings):ti,ab,kw | 56462 |
| #2 | MeSH descriptor: [Cost Savings] explode all trees | 619 |
| #3 | MeSH descriptor: [Costs and Cost Analysis] explode all trees | 16758 |
| #4 | MeSH descriptor: [Cost-Effectiveness Analysis] explode all trees | 148 |
| #5 | (economic evaluation):ti,ab,kw OR (economic analys*):ti,ab,kw OR (cost analys*):ti,ab,kw OR (cost effective* analys*):ti,ab,kw OR (cost-effective* analys*):ti,ab,kw | 50197 |
| #6 | (cost benefit* analys*):ti,ab,kw OR (cost utility* analys*):ti,ab,kw OR (cost-benefit* analys*):ti,ab,kw OR (cost-utility* analys*):ti,ab,kw OR (cost):ti,ab,kw | 77187 |
| #7 | (Cost Savings):ti,ab,kw OR (Cost Analysis):ti,ab,kw OR (cost-effectiveness):ti,ab,kw OR (economic evaluation):ti,ab,kw OR (cost-benefit):ti,ab,kw | 53864 |
| #8 | (cost-utility analysis):ti,ab,kw | 2568 |
| #9 | #1 OR #2 OR #3 OR #4 OR #5 OR #6 OR #7 OR #8 | 90151 |
| #10 | MeSH descriptor: [Infant, Extremely Premature] explode all trees | 460 |
| #11 | MeSH descriptor: [Premature Birth] explode all trees | 2562 |
| #12 | MeSH descriptor: [Infant, Premature] explode all trees | 5949 |
| #13 | MeSH descriptor: [Infant, Very Low Birth Weight] explode all trees | 1426 |
| #14 | MeSH descriptor: [Infant, Extremely Low Birth Weight] explode all trees | 184 |
| #15 | (infant premature):ti,ab,kw OR (infants premature):ti,ab,kw OR (premature infant):ti,ab,kw OR (preterm infants):ti,ab,kw OR (infant preterm):ti,ab,kw | 17669 |
| #16 | (preterm infant):ti,ab,kw OR (premature infants):ti,ab,kw OR (neonatal prematurity):ti,ab,kw OR (prematurity neonate):ti,ab,kw OR (premature infant*):ti,ab,kw | 16252 |
| #17 | (preterm infant*):ti,ab,kw OR (low birth weight):ti,ab,kw OR (low birthweight*):ti,ab,kw OR (VLBW):ti,ab,kw OR (LBW):ti,ab,kw | 19160 |
| #18 | #10 OR #11 OR #12 OR #13 OR #14 OR #15 OR #16 OR #17 | 23851 |
| #19 | MeSH descriptor: [Milk, Human] explode all trees | 1539 |
| #20 | MeSH descriptor: [Breast Milk Expression] explode all trees | 48 |
| #21 | MeSH descriptor: [Breast Feeding] explode all trees | 2981 |
| #22 | (milk human):ti,ab,kw OR (breast milk):ti,ab,kw OR (milk breast):ti,ab,kw OR (human milk):ti,ab,kw | 8713 |
| #23 | #19 OR #20 OR #21 OR #22 | 10510 |
| #24 | #9 AND #18 AND #23 | 122 |

| **Embase** | | |
| --- | --- | --- |
| **No** | Query | Results |
| #1 | 'cost'/exp OR 'cost benefit analysis'/exp OR 'cost of illness'/exp OR 'cost effectiveness analysis'/exp OR 'cost control'/exp | 689774 |
| #2 | 'costs':ti,ab,kw OR 'cost analysis':ti,ab,kw OR 'cost-benefit analysis':ti,ab,kw OR 'cost of illness':ti,ab,kw OR 'cost-effectiveness analysis':ti,ab,kw OR 'cost savings':ti,ab,kw | 445297 |
| #3 | #1 OR #2 | 906626 |
| #4 | 'extremely premature birth'/exp OR 'prematurity'/exp OR 'very low birth weight'/exp OR 'extremely low birth weight'/exp OR 'very premature birth'/exp | 157123 |
| #5 | 'extremely premature birth':ti,ab,kw OR 'premature birth':ti,ab,kw OR 'infant, premature':ti,ab,kw OR 'infant, very low birth weight':ti,ab,kw OR 'infant, extremely low birth weight':ti,ab,kw | 8977 |
| #6 | #4 OR #5 | 159759 |
| #7 | 'breast milk'/exp OR 'breast milk expression'/exp OR 'breast feeding'/exp | 99166 |
| #8 | 'milk, human':ti,ab,kw OR 'breast milk expression':ti,ab,kw OR 'breast feeding':ti,ab,kw | 19058 |
| #9 | #7 OR #8 | 102580 |
| #10 | #3 AND #6 AND #9 | 279 |

| **Web of Science Core Collection** | | |
| --- | --- | --- |
| **No** | Search | Results |
| #1 | ((((TS=(Costs and Cost Analysis)) OR TS=(Cost-Benefit Analysis)) OR TS=(Cost of Illness)) OR TS=(Cost-Effectiveness Analysis)) OR TS=(Cost Savings) | 738,867 |
| #2 | (((((((((((((((TS=(economic evaluation)) OR TS=(economic analys*)) OR TS=(cost analys*)) OR TS=(cost effective* analys*)) OR TS=(cost-effective* analys*)) OR TS=(cost benefit* analys*)) OR TS=(cost utility* analys*)) OR TS=(cost-benefit* analys*)) OR TS=(cost-utility* analys*)) OR TS=(Cost)) OR TS=(Cost Savings)) OR TS=(Cost Analysis)) OR TS=(cost-effectiveness)) OR TS=(economic evaluation)) OR TS=(cost-benefit)) OR TS=(cost-utility analysis) | 2,803,450 |
| #3 | #1 OR #2 | 2,803,450 |
| #4 | ((((TS=(infant, extremely premature)) OR TS=(Premature Birth)) OR TS=(infant, premature)) OR TS=(Infant, Very Low Birth Weight)) OR TS=(Infant, Extremely Low Birth Weight) | 63,546 |
| #5 | ((((((((((((((((TS=(infant premature)) OR TS=(infants premature)) OR TS=(premature infant)) OR TS=(preterm infants)) OR TS=(infant preterm)) OR TS=(infants preterm)) OR TS=(preterm infant)) OR TS=(premature infants)) OR TS=(neonatal prematurity)) OR TS=(prematurity neonatal)) OR TS=(premature infant*)) OR TS=(prematurity infant*)) OR TS=(preterm infant*)) OR TS=(low birth weight)) OR TS=(low birthweight)) OR TS=(VLBW)) OR TS=(LBW) | 167,606 |
| #6 | #4 OR #5 | 176,285 |
| #7 | ((TS=(milk, human)) OR TS=(Breast Milk Expression)) OR TS=(Breast Feeding) | 80,741 |
| #8 | (((TS=(milk human)) OR TS=(breast milk)) OR TS=(milk breast)) OR TS=(human milk) | 68,470 |
| #9 | #7 OR #8 | 89,433 |
| #10 | #3 AND #6 AND #9 | 308 |

Supplementary File S2. Data items extracted from included studies

Each reviewer collected the following data items from each study:

1. Year of publication

2. Publication type

3. Study period

4. Aim of the study

5. Study design

6. Population

7. Setting

8. Country

9. Inclusion criteria and exclusion criteria

10. Sample size

11. Intervention

12.Comparison

13.Outcome definition

14. Statistical methods used for analysis

15. Gestational age

16. Sex

17. Birth weight

18. Author’s Conclusions

19. Measure of health benefit

20. Cost components

21. Cost savings

22. Perspective

23. Time Horizon

24. Discount Rate

25. Evaluation type

26. Currency

27. Price year

28. Trial or model

29. Data Source

30. Type of Sensitivity Analysis

Supplementary File S3. List of references with final exclusion reasons

| No. | Studies | First author | Reasons of exclusion | Publication year |
| --- | --- | --- | --- | --- |
| 1 | Economic Analysis of Exclusive Human Milk Diets for High-Risk Neonates, a Canadian Hospital Perspective | van Katwyk S | Non-eligible population | 2020 |
| 2 | Prioritising allocation of donor human breast milk amongst very low birthweight infants in middle-income countries. | Taylor C | No relevant outcome | 2018 |
| 3 | Modelling the cost-effectiveness of human milk and breastfeeding in preterm infants in the United Kingdom. | Mahon J | Non-eligible population | 2016 |
| 4 | Mother's breast milk supplemented with donor milk reduces hospital and health service usage costs in low-birthweight infants | Dritsakou K | Non-eligible population | 2016 |
| 5 | The cost-effectiveness of using banked donor milk in the neonatal intensive care unit: prevention of necrotizing enterocolitis | Arnold LD | Non-eligible population | 2002 |

Supplementary File S4. Pediatric Quality Appraisal Questionnaire (PQAQ) of Economic Evaluations of Vision Screening Strategies in Children under the age of Six Years

| PQAQ Domain | PQAQ Score (0-1) | | | | | | | | | | | | | |
| --- | --- | --- | --- | --- | --- | --- | --- | --- | --- | --- | --- | --- | --- | --- |
|  | Tetarbe et al. (2024) | Johnson et al. (2022) | Hanford et al. (2021) | Johnson et al. (2020) | Scholz et al. (2019) | Hampson et al. (2019) | Trang et al. (2018) | Patel et al. (2017) | Assad et al. (2015) | Johnson et al. (2015) | Patel et al. (2013) | Parker et al. (2012) | Ganapathyet al. (2012) | Colaizy et al. (2016) |
| **Economic Evaluation** |  |  |  |  |  |  |  |  |  |  |  |  |  |  |
| 1. Is the research question posed in terms of costs and consequences? | 1 | 1 | 1 | 1 | 1 | 1 | 1 | 1 | 1 | 1 | 1 | 1 | 1 | 1 |
| 2. Is a specific type of economic analysis technique performed? | 1 | 1 | 1 | 1 | 1 | 1 | 1 | 1 | 1 | 1 | 1 | 1 | 1 | 1 |
| **Comparators** |  |  |  |  |  |  |  |  |  |  |  |  |  |  |
| 4. Is there a rationale for choosing the intervention(s) being investigated? | 1 | 0 | 1 | 1 | 1 | 1 | 1 | 1 | 1 | 1 | 1 | 1 | 1 | 1 |
| 5. Is there a rationale for choosing the alternative program(s) or intervention(s) used for comparison? | 1 | 0 | 1 | 1 | 1 | 1 | 1 | 0 | 0 | 0 | 0 | 1 | 1 | 1 |
| 6. Does the report describe the alternatives in adequate detail? | 1 | 0 | 1 | 1 | 1 | 1 | 1 | 0 | 0 | 0 | 0 | 1 | 1 | 1 |
| 7. Is a description of the event pathway provided? | 0 | 0 | 0 | 0 | 0 | 0 | 0 | 0 | 0 | 0 | 0 | 0 | 0 | 0 |
| 8. Is a formal decision analysis performed? | 0 | 0 | 0 | 0 | 1 | 1 | 0 | 0 | 0 | 0 | 0 | 0 | 0 | 1 |
| **Target population** |  |  |  |  |  |  |  |  |  |  |  |  |  |  |
| 9. Is the target population for the intervention identified? | 1 | 1 | 1 | 1 | 1 | 1 | 1 | 1 | 1 | 1 | 1 | 1 | 1 | 1 |
| 10. Are the subject’s representative of the population to which the intervention is targeted? | 1 | 1 | 1 | 1 | 1 | 1 | 1 | 1 | 1 | 1 | 1 | 1 | 1 | 1 |
| **Time horizon** |  |  |  |  |  |  |  |  |  |  |  |  |  |  |
| 11. Is there a time horizon for both costs and outcome? | 1 | 1 | 1 | 1 | 1 | 1 | 1 | 1 | 1 | 1 | 1 | 1 | 1 | 1 |
| 12. Do the authors justify the time horizon selected? | 1 | 1 | 1 | 1 | 1 | 1 | 1 | 1 | 1 | 1 | 1 | 1 | 1 | 1 |
| **Perspective** |  |  |  |  |  |  |  |  |  |  |  |  |  |  |
| 13. Is a perspective for the analysis given? | 1 | 1 | 1 | 1 | 1 | 1 | 1 | 1 | 1 | 1 | 1 | 1 | 1 | 1 |
| 14. Is a societal perspective taken, either alone or in addition to other perspectives? | 0 | 0 | 0 | 0 | 1 | 0 | 1 | 0 | 0 | 0 | 0 | 0 | 0 | 0 |
| 15. When there was more than one perspective, are the results of each perspective presented separately? | n/a | n/a | n/a | n/a | 0 | n/a | 0 | n/a | n/a | n/a | n/a | n/a | n/a | n/a |
| **Costs and resource use** |  |  |  |  |  |  |  |  |  |  |  |  |  |  |
| 16. Are all relevant costs for each alternative included? | 0 | 0 | 0 | 1 | 0 | 1 | 1 | 0 | 0 | 0 | 0 | 1 | 1 | 1 |
| 17. Are opportunity costs of lost time (productivity costs) for parents and informal caregivers measured when required? | n/a | n/a | n/a | n/a | n/a | n/a | n/a | n/a | n/a | n/a | n/a | n/a | n/a | n/a |
| 18. Do cost item identification and valuation extend beyond the health care system to include school and community resources when necessary? | 0 | 0 | 0 | 0 | 0 | 0 | 0 | 0 | 0 | 0 | 0 | 0 | 0 | 0 |
| 19. Are future salary and productivity changes of the child taken into consideration when appropriate? | n/a | n/a | n/a | n/a | n/a | n/a | n/a | n/a | n/a | n/a | n/a | n/a | n/a | n/a |
| 20. Were all of the sources for estimating the volume or resource use described? | 1 | 1 | 1 | 1 | 1 | 1 | 1 | 1 | 1 | 1 | 1 | 1 | 1 | 1 |
| 21. Were all the sources for estimating all of the unit costs described? | 1 | 1 | 1 | 1 | 1 | 1 | 1 | 1 | 1 | 1 | 1 | 1 | 1 | 1 |
| **Outcomes** |  |  |  |  |  |  |  |  |  |  |  |  |  |  |
| 22. Is a primary health outcome given? | 1 | 1 | 1 | 1 | 1 | 1 | 1 | 1 | 1 | 1 | 1 | 1 | 1 | 1 |
| 23. Do the authors justify the health outcomes selected? | 1 | 1 | 1 | 1 | 1 | 1 | 1 | 1 | 1 | 1 | 1 | 1 | 1 | 1 |
| 24. Is effectiveness, rather than efficacy assessed? | 1 | 1 | 1 | 1 | 1 | 1 | 1 | 1 | 1 | 1 | 1 | 1 | 1 | 1 |
| 26. Are the details of the design of the effectiveness/efficacy study(s) provided? | 1 | 1 | 1 | 1 | 1 | 1 | 1 | 1 | 1 | 1 | 1 | 1 | 1 | 1 |
| 1. Are the results of the efficacy/   effectiveness of alternatives reported? | 1 | 0 | 0 | 1 | 1 | 1 | 1 | 1 | 1 | 1 | 1 | 1 | 1 | 1 |
| 28. Are school/day care absences taken into consideration? | n/a | n/a | n/a | n/a | n/a | n/a | n/a | n/a | n/a | n/a | n/a | n/a | n/a | n/a |
| 29. If intermediate outcome variables are used, are they linked by evidence or reference to the end benefit? | n/a | n/a | n/a | n/a | n/a | n/a | n/a | n/a | n/a | n/a | n/a | n/a | n/a | n/a |
| **Analysis** |  |  |  |  |  |  |  |  |  |  |  |  |  |  |
| 33. Were costs and outcomes measured in units appropriate for the indicated analytic technique? | 1 | 1 | 1 | 1 | 0 | 1 | 1 | 1 | 1 | 1 | 1 | 1 | 1 | 1 |
| 37. Were costs valued appropriately? | 1 | 1 | 0 | 1 | 1 | 1 | 1 | 1 | 1 | 1 | 1 | 1 | 1 | 1 |
| 38. Was the valuation of outcomes appropriate for the type of analysis | 1 | 1 | 0 | 1 | 1 | 1 | 1 | 1 | 1 | 1 | 1 | 1 | 1 | 1 |
| 40. Were quantities of resources used reported separately from their unit costs? | 1 | 1 | 0 | 1 | 1 | 1 | 1 | 1 | 1 | 1 | 1 | 1 | 1 | 1 |
| 41. Were the costs aggregated correctly? | 1 | 1 | 0 | 1 | 1 | 1 | 1 | 1 | 1 | 1 | 1 | 1 | 1 | 1 |
| 42. Were details of statistical tests and confidence intervals given for stochastic data? | n/a | n/a | n/a | n/a | n/a | n/a | n/a | n/a | n/a | n/a | n/a | n/a | n/a | n/a |
| **Discounting** |  |  |  |  |  |  |  |  |  |  |  |  |  |  |
| 43. When required, were costs and consequences that occur over more than one year discounted to their present value? | 0 | 0 | 0 | 0 | 1 | 1 | 0 | 0 | 0 | 0 | 0 | 0 | 0 | 0 |
| n/a44. If costs or benefits were not discounted when the time horizon exceeded 1 year, was an explanation provided? | 0 | 0 | 0 | 0 | n/a | n/a | 0 | 0 | 0 | 0 | 0 | 0 | 0 | 0 |
| **Incremental Analysis** |  |  |  |  |  |  |  |  |  |  |  |  |  |  |
| 45. Are incremental estimates of costs and outcomes presented? | 0 | 1 | 1 | 1 | 1 | 1 | 1 | 1 | 1 | 1 | 1 | 1 | 1 | 1 |
| 46. Are the incremental estimates summarized as incremental ratios? | 0 | 0 | 0 | 0 | 1 | 0 | 1 | 0 | 0 | 0 | 1 | 0 | 0 | 0 |
| 47. Were confidence intervals/limits calculated for incremental ratios or incremental estimates of costs and outcomes? | 0 | 1 | 1 | 1 | 1 | 0 | 1 | 1 | 0 | 0 | 1 | 0 | 1 | 0 |
| **Sensitivity analysis** |  |  |  |  |  |  |  |  |  |  |  |  |  |  |
| 48. Were all important assumptions given? | 1 | 1 | 1 | 1 | 1 | 1 | 1 | 1 | 1 | 1 | 1 | 1 | 1 | 1 |
| 49. Was a sensitivity analysis performed? | 0 | 0 | 1 | 1 | 1 | 1 | 1 | 0 | 0 | 0 | 0 | 0 | 1 | 0 |
| 50. Do the authors justify the alternative values of ranges for sensitivity analysis? | 0 | 0 | 1 | 1 | 1 | 1 | 1 | 0 | 0 | 0 | 0 | 0 | 1 | 0 |
| **Conflict of Interest** |  |  |  |  |  |  |  |  |  |  |  |  |  |  |
| 52. Does the paper present the relationship with the sponsor of the study? | 1 | 1 | 0 | 0 | 1 | 1 | 1 | 1 | 0 | 1 | 1 | 0 | 1 | 1 |
| 53. Does the paper indicate that the authors had independent control over the methods and right to publish. | 1 | 1 | 1 | 1 | 1 | 1 | 1 | 1 | 1 | 1 | 1 | 1 | 1 | 1 |
| Conclusions |  |  |  |  |  |  |  |  |  |  |  |  |  |  |
| 54. Is the answer to the study question provided? | 1 | 1 | 1 | 1 | 1 | 1 | 1 | 1 | 1 | 1 | 1 | 1 | 1 | 1 |
| 55. Are the most important limitations of the study discussed? | 1 | 1 | 1 | 1 | 1 | 0 | 1 | 1 | 0 | 0 | 1 | 1 | 0 | 1 |
| 56. Do the authors generalize the conclusions to the other settings or patient/client groups? | 0 | 0 | 0 | 0 | 0 | 1 | 1 | 1 | 0 | 0 | 1 | 0 | 0 | 0 |
| 57. Global impression of the quality of the paper 1. Excellent, 2. Very Good, 3. Good, 4. Fair, 5. Poor, 6. Worthless | Good | Very Good | Good | Very Good | Very Good | Good | Good | Good | Good | Good | Excellent | Fair | Good | Good |
| Overall quality assessment | moderate(27/46) | moderate(25/46) | moderate(25/46) | moderate(31/46) | moderate(34/46) | moderate(33/46) | high (35/46) | moderate(28/46) | moderate(24/46) | moderate(25/46) | moderate(29/46) | moderate(28/46) | moderate(31/46) | moderate(29/46) |

Note:

Each item was scored as fully meeting the criteria (“1”), not meeting them at all (“0”),n/a not applicable. Three quality categories were established for the **PQAQ** score, with a maximum score of 46: high (>75%), moderate (50-74%), and low (<50%)

Supplementary File S5. Completed PRISMA checklist

| **Section and Topic** | **Item #** | **Checklist item** | **Location where item is reported** |
| --- | --- | --- | --- |
| **TITLE** | | |  |
| Title | 1 | Identify the report as a systematic review. | 1 |
| **ABSTRACT** | | |  |
| Abstract | 2 | See the PRISMA 2020 for Abstracts checklist. | 1 |
| **INTRODUCTION** | | |  |
| Rationale | 3 | Describe the rationale for the review in the context of existing knowledge. | 2-3 |
| Objectives | 4 | Provide an explicit statement of the objective(s) or question(s) the review addresses. | 3 |
| **METHODS** | | |  |
| Eligibility criteria | 5 | Specify the inclusion and exclusion criteria for the review and how studies were grouped for the syntheses. | 3-4 |
| Information sources | 6 | Specify all databases, registers, websites, organisations, reference lists and other sources searched or consulted to identify studies. Specify the date when each source was last searched or consulted. | 3 |
| Search strategy | 7 | Present the full search strategies for all databases, registers and websites, including any filters and limits used. | 3 |
| Selection process | 8 | Specify the methods used to decide whether a study met the inclusion criteria of the review, including how many reviewers screened each record and each report retrieved, whether they worked independently, and if applicable, details of automation tools used in the process. | 4 |
| Data collection process | 9 | Specify the methods used to collect data from reports, including how many reviewers collected data from each report, whether they worked independently, any processes for obtaining or confirming data from study investigators, and if applicable, details of automation tools used in the process. | 5 |
| Data items | 10a | List and define all outcomes for which data were sought. Specify whether all results that were compatible with each outcome domain in each study were sought (e.g. for all measures, time points, analyses), and if not, the methods used to decide which results to collect. | 4-5 |
|  | 10b | List and define all other variables for which data were sought (e.g. participant and intervention characteristics, funding sources). Describe any assumptions made about any missing or unclear information. | 4-5 |
| Study risk of bias assessment | 11 | Specify the methods used to assess risk of bias in the included studies, including details of the tool(s) used, how many reviewers assessed each study and whether they worked independently, and if applicable, details of automation tools used in the process. | 5 |
| Effect measures | 12 | Specify for each outcome the effect measure(s) (e.g. risk ratio, mean difference) used in the synthesis or presentation of results. | 5 |
| Synthesis methods | 13a | Describe the processes used to decide which studies were eligible for each synthesis (e.g. tabulating the study intervention characteristics and comparing against the planned groups for each synthesis (item #5)). | - |
|  | 13b | Describe any methods required to prepare the data for presentation or synthesis, such as handling of missing summary statistics, or data conversions. | - |
|  | 13c | Describe any methods used to tabulate or visually display results of individual studies and syntheses. | - |
|  | 13d | Describe any methods used to synthesize results and provide a rationale for the choice(s). If meta-analysis was performed, describe the model(s), method(s) to identify the presence and extent of statistical heterogeneity, and software package(s) used. | - |
|  | 13e | Describe any methods used to explore possible causes of heterogeneity among study results (e.g. subgroup analysis, meta-regression). | - |
|  | 13f | Describe any sensitivity analyses conducted to assess robustness of the synthesized results. | - |
| Reporting bias assessment | 14 | Describe any methods used to assess risk of bias due to missing results in a synthesis (arising from reporting biases). | - |
| Certainty assessment | 15 | Describe any methods used to assess certainty (or confidence) in the body of evidence for an outcome. | - |
| **RESULTS** | | |  |
| Study selection | 16a | Describe the results of the search and selection process, from the number of records identified in the search to the number of studies included in the review, ideally using a flow diagram. | 5 |
|  | 16b | Cite studies that might appear to meet the inclusion criteria, but which were excluded, and explain why they were excluded. | 5 |
| Study characteristics | 17 | Cite each included study and present its characteristics. | 5 |
| Risk of bias in studies | 18 | Present assessments of risk of bias for each included study. |  |
| Results of individual studies | 19 | For all outcomes, present, for each study: (a) summary statistics for each group (where appropriate) and (b) an effect estimate and its precision (e.g. confidence/credible interval), ideally using structured tables or plots. | 5-8 |
| Results of syntheses | 20a | For each synthesis, briefly summarise the characteristics and risk of bias among contributing studies. | - |
|  | 20b | Present results of all statistical syntheses conducted. If meta-analysis was done, present for each the summary estimate and its precision (e.g. confidence/credible interval) and measures of statistical heterogeneity. If comparing groups, describe the direction of the effect. | - |
|  | 20c | Present results of all investigations of possible causes of heterogeneity among study results. | - |
|  | 20d | Present results of all sensitivity analyses conducted to assess the robustness of the synthesized results. | - |
| Reporting biases | 21 | Present assessments of risk of bias due to missing results (arising from reporting biases) for each synthesis assessed. | - |
| Certainty of evidence | 22 | Present assessments of certainty (or confidence) in the body of evidence for each outcome assessed. | - |
| **DISCUSSION** | | |  |
| Discussion | 23a | Provide a general interpretation of the results in the context of other evidence. | 8 |
|  | 23b | Discuss any limitations of the evidence included in the review. | 11 |
|  | 23c | Discuss any limitations of the review processes used. | 11 |
|  | 23d | Discuss implications of the results for practice, policy, and future research. | 11 |
| **OTHER INFORMATION** | | |  |
| Registration and protocol | 24a | Provide registration information for the review, including register name and registration number, or state that the review was not registered. | 3 |
|  | 24b | Indicate where the review protocol can be accessed, or state that a protocol was not prepared. | 3 |
|  | 24c | Describe and explain any amendments to information provided at registration or in the protocol. | 3 |
| Support | 25 | Describe sources of financial or non-financial support for the review, and the role of the funders or sponsors in the review. | 12 |
| Competing interests | 26 | Declare any competing interests of review authors. | 12 |
| Availability of data, code and other materials | 27 | Report which of the following are publicly available and where they can be found: template data collection forms; data extracted from included studies; data used for all analyses; analytic code; any other materials used in the review. | 15-16 |

*From:*  Page MJ, McKenzie JE, Bossuyt PM, Boutron I, Hoffmann TC, Mulrow CD, et al. The PRISMA 2020 statement: an updated guideline for reporting systematic reviews. BMJ 2021;372:n71. doi: 10.1136/bmj.n71

For more information, visit: <http://www.prisma-statement.org/>
